# Reshaping the path of mild cognitive impairment by refining exercise prescription: A trial designed to understand the type of exercise, for whom, and how

**DOI:** 10.1002/alz70860_102757

**Published:** 2025-12-23

**Authors:** Teresa Liu‐Ambrose

**Affiliations:** ^1^ Vancouver Coastal Health Research Institute, Vancouver, BC, Canada; Centre for Aging SMART, Vancouver Coastal Health Research Institute, Vancouver, BC, Canada; University of British Columbia, Vancouver, BC, Canada; Djavad Mowafaghian Centre for Brain Health, Vancouver, BC, Canada

## Abstract

**Background:**

Targeted exercise training is a promising strategy for promoting cognitive function and preventing dementia in older age. Despite the utility of exercise as an intervention, variation still exists in exercise‐induced cognitive gains and questions remain regarding the type of training (*i.e*., what), as well as moderators (*i.e*., for whom) and mechanisms (*i.e*., how) of benefit. Both aerobic training (AT) and resistance training (RT) enhance cognitive function in older adults without cognitive impairment; however, the vast majority of trials have focused exclusively on AT. Thus, more research is needed on RT, as well as on the combination of AT and RT, in older adults with mild cognitive impairment (MCI), a prodromal stage for dementia. Therefore, we conducted a 6‐month, 2x2 factorial randomized controlled trial in older adults with MCI to assess the individual effects of AT and RT, as well as the combined effect of AT and RT on cognitive function and to determine the possible underlying biological mechanisms.

**Methods:**

226 community‐dwelling adults, aged 65 to 85 years, with MCI from metropolitan Vancouver were recruited to participate in this study. Randomization was stratified by biological sex and participants were randomly allocated to one of the four experimental groups: 1) 4x/week balance and tone (BAT; *i.e*., active control); 2) combined 2x/week AT + 2x/week RT; 3) 2x/week AT + 2x/week BAT; or 4) 2x/week RT + 2x/week BAT. The primary outcome was Alzheimer's Disease Assessment Scale‐Cognitive‐Plus. Secondary outcomes included cognitive function, health related quality of life, physical function, actigraphy measures, questionnaires, and falls. Outcomes were measured at baseline, 6 months (*i.e*., trial completion), and 18 months (*i.e*., 12‐month follow‐up).

**Discussion:**

Establishing the efficacy of different types and combinations of exercise training to minimize cognitive decline will advance our ability to prescribe exercise as “medicine” to treat MCI and delay the onset and progression of dementia. This trial provides insight as to type of exercise training (what) may be most beneficial for MCI, for whom (i.e., sex differences) with MCI, and how.

